# Sequencing SARS-CoV-2 genomes from saliva

**DOI:** 10.1093/ve/veab098

**Published:** 2022-01-03

**Authors:** Tara Alpert, Chantal B F Vogels, Mallery I Breban, Mary E Petrone, Anne L Wyllie, Nathan D Grubaugh, Joseph R Fauver

**Affiliations:** Department of Epidemiology of Microbial Diseases, Yale School of Public Health, 60 College St, New Haven, CT 06510, USA; Department of Epidemiology of Microbial Diseases, Yale School of Public Health, 60 College St, New Haven, CT 06510, USA; Department of Epidemiology of Microbial Diseases, Yale School of Public Health, 60 College St, New Haven, CT 06510, USA; Department of Epidemiology of Microbial Diseases, Yale School of Public Health, 60 College St, New Haven, CT 06510, USA; Department of Epidemiology of Microbial Diseases, Yale School of Public Health, 60 College St, New Haven, CT 06510, USA; Department of Ecology and Evolutionary Biology, Yale University, 165 Prospect St., New Haven, CT 06511, USA; Department of Epidemiology of Microbial Diseases, Yale School of Public Health, 60 College St, New Haven, CT 06510, USA

**Keywords:** SARS-CoV-2; saliva, next generation sequencing, genomic epidemiology, oxford nanopore MinION, salivadirect

## Abstract

Genomic sequencing is crucial to understanding the epidemiology and evolution of Severe acute respiratory syndrome coronavirus 2 (SARS-CoV-2). Often, genomic studies rely on remnant diagnostic material, typically nasopharyngeal (NP) swabs, as input into whole-genome SARS-CoV-2 next-generation sequencing pipelines. Saliva has proven to be a safe and stable specimen for the detection of SARS-CoV-2 RNA via traditional diagnostic assays; however, saliva is not commonly used for SARS-CoV-2 sequencing. Using the ARTIC Network amplicon-generation approach with sequencing on the Oxford Nanopore MinION, we demonstrate that sequencing SARS-CoV-2 from saliva produces genomes comparable to those from NP swabs, and that RNA extraction is necessary to generate complete genomes from saliva. In this study, we show that saliva is a useful specimen type for genomic studies of SARS-CoV-2.

## 1. Introduction

Genomic studies of SARS-CoV-2 are critical to the collective understanding and control of the coronavirus disease 2019 (COVID-19) pandemic. Unbiased genomic sequencing identified a novel betacoronavirus (SARS-CoV-2) in the bronchoalveolar lavage fluid of an individual with pneumonia in December, 2019 (Wu et al. 2020). This first SARS-CoV-2 genome sequence paved the way for the design of multiple vaccines (Corbett et al. 2020), the development of diagnostic assays (Vogels et al. 2020), and targeted sequencing approaches (Quick 2020). Since then, more than 5 million SARS-CoV-2 consensus sequence genomes have been uploaded and shared on GISAID (gisaid.org) as of 21 June 2021. Open data sharing has facilitated wide-scale viral surveillance that has led to the identification of multiple variants of concern and shaped both clinical and public health approaches to the treatment and control of COVID-19 (Grubaugh et al. 2021).

Workflows for whole-genome SARS-CoV-2 sequencing begin with sample collection, typically from discarded clinical diagnostic specimens. While nasopharyngeal (NP) swabs are still considered as the ‘gold standard’ for SARS-CoV-2 diagnostic testing, we have shown that saliva is a sensitive (Wyllie et al. 2020; Watkins et al. 2021) and stable (Ott et al. 2021) sample type, which can be reliably self-collected (Petrone et al. 2021), for the detection of SARS-CoV-2 RNA by Reverse Transcription Quantitative Polymerase Chain Reaction (RT-qPCR) diagnostic assays in the absence of RNA extraction (Vogels et al. 2021b). As saliva is increasingly being used in diagnostic testing programs, we sought to determine if saliva samples can be used to generate high-quality SARS-CoV-2 genomes.

In this study, we consider two common measures of genome quality: depth of coverage (i.e. the number of reads aligning to the genome per genome position) and breadth of coverage (i.e. genome completeness). Viral diversity, polymerase errors, and sequencing artifacts introduce heterogeneity into sequencing data, making it difficult to determine the consensus nucleotide identity with few reads. The ARTIC Network protocol only assigns a nucleotide identity to positions with at least 20× coverage (i.e. 20 or more reads align to a given position in the genome) to increase confidence in the consensus genome sequence. The completeness of a sequenced SARS-CoV-2 genome often depends on the viral load and sample quality. Because SARS-CoV-2 RNA typically makes up a small proportion of RNA in clinical diagnostic specimens, PCR amplification increases the amount of genomic material available for sequencing. However, due to the highly multiplexed nature of the ARTIC Network PCR approach, where more than a hundred individual primer sets are pooled in a single reaction, a slight imbalance in priming efficiency can lead to unequal read distribution and result in incomplete genomes. Thus, genome completeness is rarely 100 per cent, rather genomes that are 80–99 per cent complete are considered high quality for the purposes of this study.

**Figure 1.**
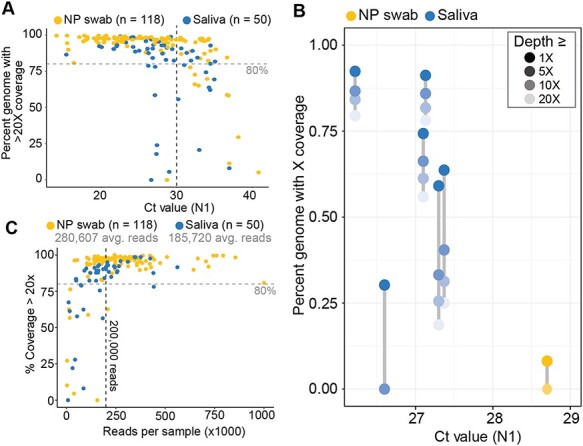
Saliva performs comparably to nasopharyngeal (NP) swabs as an original sample for SARS-CoV-2 genome sequencing. (A) The percent of genome with at least 20× coverage is plotted against the Ct value for the N1 target for a cohort of unpaired saliva (blue) and NP swab (yellow) samples. Samples with a Ct value ≤30 (vertical black dashed line) and a genome completeness <80 per cent (horizontal grey line) are displayed in panel B. (B) The percent of the genome at different coverage thresholds (legend, top right) is plotted against Ct value for the N1 target for select samples from A. Grey lines connect points related to the same sample. (C) A subset of samples from the cohorts in panel A are plotted against the number of reads for each sample, showing that nearly all samples (saliva and NP swab) with at least 200,000 reads (vertical black line) have >80 per cent genome completeness. The mean readcount for each cohort is displayed underneath the legend.

We compared SARS-CoV-2 genome quality from (1) saliva and NP swabs of varying viral concentrations from a hospital cohort, (2) paired saliva and NP swab samples from the same individual, and (3) saliva samples with and without RNA extraction (i.e. the isolation and purification of total RNA from saliva and NP swabs). Our results show that saliva performs similarly to NP swabs using both random and paired samples, genome completeness is strongly correlated with viral load and data quantity, and that performing RNA extraction from saliva drastically improves genome completeness. These data demonstrate that high-quality SARS-CoV-2 genomes can be readily sequenced from saliva samples.

## 2. Results

### 2.1 NP swabs and saliva samples produce high-quality SARS-CoV-2 genomes

To establish whether we could generate whole SARS-CoV-2 genomes from saliva, we used the ARTIC Network amplicon generation approach to sequence SARS-CoV-2 RNA extracted from 118 NP swabs and 50 saliva samples on the Oxford Nanopore MinION. These samples represent a random subset of SARS-CoV-2 genomes generated as a part of the Yale Biorepository that encompass a range of Cycle Threshold (CT) values and are not matched from the same individual. We found that the cycle threshold (Ct) value for the N1 target according to the Centers for Disease Control and Prevention (CDC) SARS-CoV-2 RT-qPCR diagnostic assay is associated with genome completeness for both NP swabs and saliva samples (Fig. 1A). Specifically, Ct values are inversely correlated with viral RNA quantity, and we could sequence more complete SARS-CoV-2 genomes from samples with low Ct values, regardless of sample type. We generated high-quality genomes from saliva samples with a wide range of Ct values. A Ct value of 30 is often used as a threshold by sequencing labs, where samples with higher Ct values may not generate complete SARS-CoV-2 genomes (Kubik et al. 2021). Based on our RT-qPCR standard curve, a Ct value of 30 corresponds to ∼1,000 SARS-CoV-2 genome equivalents (GE) per microliters (see Section 4). Our data show that we could sequence the majority of samples with a Ct ≤30 to >80 per cent completeness (117/124, 94.3 per cent) (Supplementary Table S1). However, we generated fewer genomes with >80 per cent completeness from saliva samples with a Ct ≤30 (30/36, 83.3 per cent) compared to NP swabs (87/88, 98.8 per cent).

**Figure 2.**
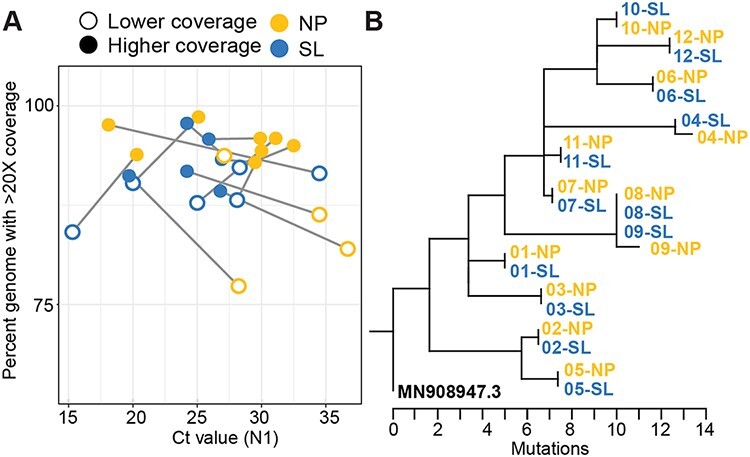
SARS-CoV-2 genomes from matched saliva and NP swabs are similar in completeness and content. (A) A cohort of matched saliva and NP swab samples from the same individual were sequenced and reads were subsampled to match the mate with fewer reads. A grey line connects the mates and an empty circle highlights the mate with lower coverage. (B) A maximum-likelihood tree of matched saliva and NP swab samples from Fig. 2A is rooted against the reference genome (NCBI Accession MN908947.3) to show pairwise identity. Tips of the tree aligning vertically indicate that the genomes from these samples are identical.

As we require ≥20 aligned sequencing reads to call a base at any genome position, it is useful to assess lower depths of coverage to determine if more complete genomes would be generated with more data. We evaluated the discrepancy in SARS-CoV-2 genome completeness between sample types by calculating genome completeness at various depths of coverage for the six saliva samples and one NP swab with a low Ct value (≤30) and low genome completeness (<80 per cent) (Fig. 1B). For the six saliva samples at 10×, 5×, or 1× depth of coverage, we found that sequencing reads were generated across the genome, suggesting that simply allocating more sequencing space per sample would have resulted in more complete genomes. However, genome completeness was only slightly improved at lower depths of coverage for the single NP swab sample. This NP swab sample appears to be an anomaly, as all other SARS-CoV-2 genomes generated from NP swabs with CTs <30 generated high-quality genomes.

We sequenced all samples included in this study in a multiplexed fashion, with as many as twenty-two samples included per run, and therefore, the total amount of sequencing space given to each sample varies. As expected, we found a direct relationship between the total number of reads per sample and the genome completeness for both saliva and NP swabs (Fig. 1C). Of the fifty-one samples composed of >200,000 reads, fifty (98 per cent) exhibited high levels of genome completeness (>80 per cent). Therefore we suggest allocating at least 200,000 reads per sample, regardless of sample type, to generate near-complete SARS-CoV-2 genomes using this sequencing approach. We have observed more variability in depth of coverage distribution from samples with higher CT values, indicating that more data are necessary to generate high-quality genomes from these samples.

### 2.2 NP swabs and saliva samples collected from the same individual produce genetically identical and complete SARS-CoV-2 genomes

Next, we examined if saliva samples are directly comparable to NP swabs by sequencing matched samples taken from the same individual at the same time point. We obtained matched samples from twelve individuals and randomly downsampled the sequencing reads to match the sample with the least amount of data. We found differences in genome completeness for different sample types collected from the same individual, although these differences were minor, were not biased toward one sample type, and all samples produced genomes >75 per cent complete (Fig. 2A). For the twelve individuals with matched SARS-CoV-2 genomes from saliva and NP swabs (Fig. 2A), NP swabs produced the more complete genome in seven of twelve individuals. Saliva samples have a mean genome completeness of 91.1 per cent ±0.04 per cent compared to NP samples at 92.0 per cent ±0.07 per cent. We did not observe obvious differences in the depth of coverage across genomes by sample type from matched specimens (Supplementary Fig. S2). Instead, we observed areas of the genome where amplicons consistently were not adequately generated and sequenced, regardless if samples came from NP swabs or saliva samples. Consistent with our previous results (Wyllie et al. 2020), we found lower SARS-CoV-2 Ct values from saliva compared to the matched NP swabs. We also compared the genetic relatedness of every matched sample by constructing a maximum-likelihood phylogenetic tree. We found that ten of the twelve pairs were 100 per cent identical across the entire genome, as indicated by vertical alignment of tips in the phylogenetic tree (Fig. 2B). In two pairs (04 and 09), the genome sequenced from the NP swab had a single additional mutation compared to the genome from saliva, as indicated by the extended branch length seen in the NP sample for both pairs. The mutations identified from pair 04 (N gene, C28854T) and pair 09 (S gene, C23271T) are well supported with >400× depth of coverage at the sites, occurred outside of primer-binding regions, and have not been identified as problematic sites for phylogenetic resolution (ProblematicSites_SARS-CoV2 2020). These mutations did not change the lineage placement for the NP swab genomes from samples 04-NP and 09-NP, both clustering with the B.1 pangolin lineage.

### 2.3 RNA extraction improves completeness of SARS-CoV-2 genomes from saliva

**Figure 3.**
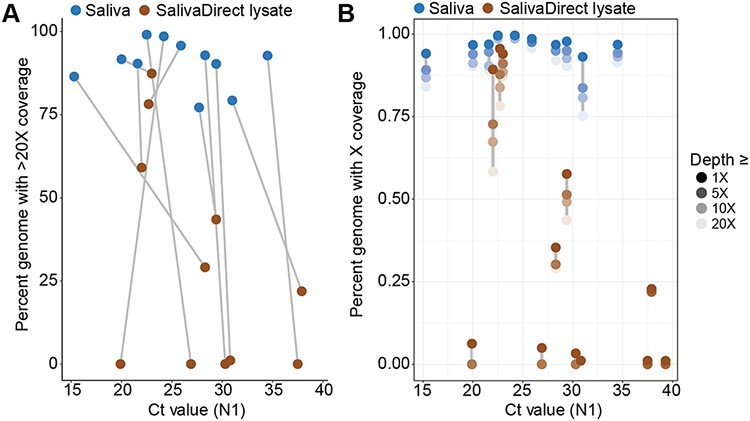
RNA extraction dramatically improves SARS-CoV-2 genome completeness from saliva samples. (A) Saliva samples were split to perform either RNA extraction (blue) or SalivaDirect lysate (brown) preparation (incubation with Proteinase K at 95°C for 5 minutes; see Section 4) and were sequenced. The percent of genome with at least 20× coverage is plotted against the Ct value for the N1 target for matched samples (connected by grey line). (B) The percent of the genome at different coverage thresholds (legend, right) is plotted against Ct value for the N1 target for the same cohort of samples in panel A. Grey lines connect points related to the same sample.

The removal of the RNA extraction step for SARS-CoV-2 diagnostic test can streamline workflows and has been demonstrated for multiple sample types, including saliva (Lübke et al. 2020; Smyrlaki et al. 2020; Vogels et al. 2021b). SARS-CoV-2 genomic sequencing workflows would similarly benefit in the time and cost reductions by removing RNA extraction steps. We attempted to sequence SARS-CoV-2 genomes from split saliva samples, where half of the sample underwent our normal RNA extraction procedure and the other half was processed according to our SalivaDirect protocol with a proteinase K and heat treatment instead of RNA extraction (Vogels et al. 2021b). While the extraction-free SalivaDirect protocol yielded similar SARS-CoV-2 Ct values to samples with RNA extraction, indicating similar levels of PCR detection, we found that sequencing from the SalivaDirect substrate led to a substantially lower genome completeness of 7.8 per cent ± 33.3 per cent compared to RNA extracted samples averaging 89.3 per cent ± 29.0 per cent completeness (Fig. 3A). In fact, of the eleven samples processed using our SalivaDirect protocol, only one sequenced genome met our standards of 80 per cent completeness, while the majority produced genomes that were less than 50 per cent complete. In comparison to the counterpart sample that did undergo RNA extraction, we sequenced nine out of eleven to >80 per cent completeness, including some samples with Ct values >30. We then investigated whether this observation could be attributed to insufficient data by calculating the percentage completeness of each genome at various levels of depth of coverage (Fig. 3B). Our analysis showed that increasing data per sample would improve genome completeness for only four of the samples processed with the extraction-free SalivaDirect protocol as evidenced by the large separation between data points in Fig. 3B. However, eight of these samples had no reads aligning to >90 per cent of the genome, indicating that more data would not have substantially improved completeness, even at low Ct values. Seven of the eleven lysates received >100,000 reads (Supplemental Data), indicating that unequal amplification accounts for incomplete genomes being generated from these samples. Thus, without further protocol optimization, RNA extract from saliva samples is necessary for sequencing complete SARS-CoV-2 genomes.

## 3. Discussion

In this study, we determined that SARS-CoV-2 genomes generated from saliva were of the same completeness as those generated from NP swabs, the gold standard diagnostic specimen for COVID-19. By comparing a random subset of samples from saliva and NP swabs spanning different viral titers (Fig. 1A), we found little difference between genome completeness from different sample types. Rather, viral titer (Ct values) and the amount of sequencing data generated from each sample are more indicative of genome completeness than specimen type (Fig. 1B, C). Our results demonstrate that samples with a Ct value at or below 30, or ∼1000 SARS-CoV-2 GE/µl, with greater than 200,000 reads should produce near-complete SARS-CoV-2 genomes using the ARTIC amplicon-based sequencing approach, in line with previously published data (Kubik et al. 2021). We observed variation in Ct values from matched saliva and NP samples (Fig. 2A); however, the genome sequence itself was identical or nearly identical regardless of sample type (Fig. 2B). When comparing saliva samples that have undergone RNA extraction to identical saliva samples processed with our SalivaDirect approach that excludes RNA extraction, we see a substantial drop in genome completeness (Fig. 3A, B). This result may be explained by the highly multiplexed nature of the amplicon generation step being less efficient when RNA is not extracted and purified, as we observed noticeably lower concentrations post-amplicon generation and unequal coverage distribution across the genome from SalivaDirect lysates. While SalivaDirect is a robust SARS-CoV-2 diagnostic approach, it is not currently optimized for generating complete SARS-CoV-2 genomes.

There are important considerations for the interpretation of these data. Our Ct value threshold of 30 is conservative and should not be seen as a lower limit of detection. Ct values will vary based on RT-qPCR assays, reagents, and thermocyclers, and we were able to sequence some near-complete SARS-CoV-2 genomes at Ct values greater than 30. However, for samples with CT values >30, more data are needed to generate high-quality genomes because depth of coverage is more variable across the genome. Because of the highly multiplexed amplicon sequencing approach, some primer pairs are more favorable and more amplicons from these pairs will be generated, which is exacerbated in samples with high CT values. Modifications of the primer concentrations used in the ARTIC Network’s amplicon-based sequencing approach have resulted in improved read distribution across the SARS-CoV-2 genome, although we have not assessed newer versions performance with saliva samples (Pipelines et al. 2020). Our data were generated prior to these modifications, thus we would expect fewer reads per sample will be needed to generate high-quality genomes. Similarly, individual laboratory validation is necessary to determine the amount of data needed per sample if using protocols outside of the specific library preparation and sequencing approaches used in this study. As well, data presented here was generated on the Oxford Nanopore MinION where the entire ∼400 b.p. amplicon is sequenced in a single read. Most library preparation approaches used for Illumina sequencing platforms fragment amplicons for shorter library insert lengths, meaning more reads will need to be generated per sample on short-read sequencing platforms.

In addition to these considerations, there are some limitations to our results. We did not assess how saliva performs as a specimen for sequencing SARS-CoV-2 genomes outside of amplicon-based strategies and additional research is needed to assess how saliva would perform in metagenomic or hybrid-/capture-based sequencing approaches. Additionally, we did not quantify the effect RNA extraction has on generating complete genomes from NP swabs. Therefore, we cannot determine if the reduction in genome completeness seen from samples without RNA extraction is a specimen-type-specific phenomenon. As well, we limited our comparisons of saliva samples to NP swabs and did not include additional common sample types for SARS-CoV-2 diagnostics, including anterior nasal and oropharyngeal swabs (Kevadiya et al. 2021).

Previous studies have shown that saliva is a stable and sensitive specimen type for SARS-CoV-2 diagnostic assays (Wyllie et al. 2020; Ott et al. 2021; Watkins et al. 2021). Ott et al. (2021) found that Ct values in saliva remained stable for up to 25 days at room temperature and through a freeze/thaw cycle. While we did not assess the ability to sequence SARS-CoV-2 genomes from saliva after long periods of storage at room temperature, previous results suggest it would be feasible. In addition to the safety afforded through self-collection opposed to NP swabs, saliva is increasingly becoming an appealing specimen type, in particular for large-scale public health surveillance. Taken together, our data indicate that saliva is a satisfactory sample type for not only diagnostic assays but also for sequencing high-quality SARS-CoV-2 genomes. This is an important finding for the scalability and streamlining of genomic epidemiological studies.

### 3.1 Conclusions

The results of our study comparing the use of saliva to NP swabs for SARS-CoV-2 genome sequencing indicate that viral titers and data quantity influence genome completeness more than specimen type.

## 4. Methods

### 4.1 Resource availability

#### 4.1.1 Lead contact

Further information and requests for resources and reagents should be directed to and will be fulfilled by the lead contact, Joseph R. Fauver (joseph.fauver@yale.edu).

#### 4.1.2 Materials availability

This study did not generate new unique reagents.

#### 4.1.3 Data and code availability

All consensus genomes used in this study have been deposited at https://github.com/josephfauver/Saliva_Sequencing_Manuscript and are publicly available as of the date of publication. Accession numbers are listed in the key resources table.

All original code has been deposited at https://github.com/josephfauver/Saliva_Sequencing_Manuscript and is publicly available as of the date of publication.

Any additional information required to reanalyze the data reported in this paper is available from the lead contact upon request.

### 4.2 Experimental model and subject details

NP swabs and saliva were collected from enrolled COVID-19 inpatients and health-care workers from Yale-New Haven Hospital in accordance with the Yale University HIC-approved protocol #2000027690. Samples were collected after the study participant had acknowledged that they had understood the study protocol and signed the informed consent. All participant information and samples were collected in association with non-individually identifiable study identifiers. These samples were used to create the Yale COVID-19 Biorepository.

### 4.3 Method details

#### 4.3.1 Sample selection and RNA extraction

Original nasal swab in viral transport media or original saliva samples were processed at the Yale School of Public Health. NP swabs and saliva samples came from the Yale-New Haven Hospital in partnership with the Yale Pathology Lab, Yale Clinical Virology Lab, as a part of the Yale COVID-19 Biorepository, explained in depth in the Supplemental Appendix of Wyllie et al. (2020). Nucleic acid was extracted from original samples (300 μl) using the MagMAX viral/pathogen nucleic acid isolation kit (Thermo Fisher) and eluted into 75 μl. Samples were screened for SARS-CoV-2 RNA via a multiplex RT-qPCR assay using the NEB Luna universal probe 1-Step RT-qPCR kit with CDC N1, N2, and RP primer-probe sets on the Bio-Rad CFX96 touch real-time PCR detection system (Kudo et al. 2020). PCR conditions were as follows: 55°C for 10 minutes, 95°C for 1 minute, 45 cycles of 95°C for 10 seconds followed by 55°C for 30 seconds. A standard curve of synthetic RNA transcripts was used to convert Ct values to GE (available on https://github.com/josephfauver/Saliva_Sequencing_Manuscript). Multiple extraction controls were included for each RNA extraction batch and tested negative for SARS-CoV-2 RNA by the same assay.

#### 4.3.2 SalivaDirect

A detailed SalivaDirect protocol has been published (Vogels et al. 2021a). Briefly, 50 μl of saliva is combined with 2.5 μl (50 mg/mL) Proteinase K (Thermo Fisher) and vortexed for 1 minute at 3,200 RPM. After 5 minutes of incubation at 95°C for 5 minutes, 5 μl of lysate was directly used as input in the RT-qPCR with the same conditions as specific for extracted RNA above. Samples were stored at −80°C before sequencing.

#### 4.3.3 Oxford Nanopore library preparation and sequencing

RNA extracted from positive samples or processed SalivaDirect samples served as the inputs for an amplicon-based approach for sequencing on the Oxford Nanopore Technologies (ONT; Oxford, United Kingdom) MinION (Quick 2020). Sequencing libraries were prepared using the ONT Ligation Sequencing Kit (SQK-LSK109) and the ONT Native Barcoding Expansion pack as described in the ARTIC Network’s protocol with V3 primers (IDT) with the following modifications: cDNA was generated with SuperScriptIV VILO Master Mix (Thermo Fisher Scientific, Waltham, MA, USA), all amplicons were generated using 35 cycles of amplification, amplicons were then normalized to 15 ng for each sample, end repair incubation time was increased to 25 minutes followed by an additional bead-based cleanup, and all cleanup steps used a ratio of 1:1 beads:sample. Normalized samples were pooled following barcode ligation and prior to adaptor ligation. No-template controls (H2O) were introduced for each run at the cDNA synthesis and amplicon generation steps and were taken through the entire library preparation and were sequenced alongside SARS-Cov-2-positive samples to detect any cross-contamination. For each control in each run, less than 1,000 total reads were observed. A subset of reads in control samples aligned to the SARS-CoV-2 genome, although no position of the genome had greater than 20 reads, i.e. enough data to influence the generation of a consensus genome. 25 ng of the final library was loaded on a MinION R9.4.1 flow cell and sequenced for approximately 8–10 hours. As more reads were required to generate complete genomes from samples with high CT values, libraries containing these samples were run for longer compared to libraries of samples with low CT values. Accordingly, run time was not standardized across sequencing runs.

#### 4.3.4 Bioinformatics processing

The RAMPART application from the ARTIC Network was used to monitor approximate genome coverage and completeness for each sample and control in real time during the sequencing run (github.com/artic-network/rampart). Fast5 files were basecalled using the Guppy basecaller 4.4.0 fast model, and consensus genomes were generated according to the ARTIC bioinformatic pipeline (artic.network/ncov-2019/ncov2019-bioinformatics-sop.html), which uses Nanopolish to call variants (Loman, Quick, and Simpson et al. 2015). A threshold of 20× coverage was required to call a base pair in the consensus genome. Regions of the genome with less than 20× coverage were designated with N’s in the consensus genome. The total number of reads from any amplicon was capped at 400 using the normalize flag in the ARTIC Networks bioinformatic pipeline. When comparing genomes generated from saliva and NP swabs collected from the same individual, we randomly downsampled the .fastq files to match the sample with the fewest reads.

#### 4.3.5 Quantification and statistical analysis

Genome completeness was determined either by counting N’s in the consensus genome output by the ARTIC pipeline for 20× coverage (total genome length minus total Ns) (Fig. 1A and C) or by using the SAMtools (Li et al. 2009) depth feature for various coverage thresholds (Fig. 1B). At the 20× threshold, these two methods produced nearly identical values, with the only discrepancies occurring in the primer binding regions. The bioinformatic pipeline masks primer binding sites to not influence consensus sequences, whereas SAMtools depth features counts all reads aligning to the genome. The differences occur in primer sequences where amplicons drop out, and are relatively minimal, representing ±1 per cent of the total genome.

Matched saliva and NP swab genomes were aligned with MAFFT (Katoh et al. 2002; Katoh and Standley 2013), and alignments were inspected prior to phylogenetic analysis to ensure correct alignment with the SARS-CoV-2 reference genome (MN908947.3), with a particular focus on regions of the sample genomes where a nucleotide could not be reliably called (<20× coverage). Amplicons tend to ‘drop-out’ in the same region across multiple samples, resulting in long stretches of NNN’s in consensus genomes and may appear as insertions in a multisequence alignment. Manual curation of alignments was conducted prior to phylogenetic analysis using a maximum-likelihood approach (PHYML) to show similarity between matched samples (Guindon et al. 2010). Lineage assignments were conducted using pangolin (v.3.1.3) (Áine O’Toole et al. 2020).

## Supplementary Material

veab098_Supp

## Yale IMPACT research team members

Kelly Anastasio, Michael H. Askenase, Maria Batsu, Santos Bermejo, Sean Bickerton, Anderson Brito, Kristina Brower, Molly L. Bucklin, Staci Cahill, Melissa Campbell, Yiyun Cao, Arnau Casanovas-Massana, Edward Courchaine, Rupak Datta, Giuseppe DeIuliis, Charles Dela Cruz, Rebecca Earnest, Shelli Farhadian, Renata Filler, John Fournier, Bertie Geng, Laura Glick, Benjamin Goldman-Israelow, Ryan Handoko, Christina Harden, Akiko Iwasaki, Cole Jensen, Chaney Kalinich, William Khoury-Hanold, Daniel Kim, Jon Klein, Jonathan Klein, Lynda Knaggs, Albert Ko, Maxine Kuang, Eriko Kudo, Sarah Lapidus, Joseph Lim, Melissa Linehan, Feimei Liu, Peiwen Lu, Alice Lu-Culligan, Carolina Lucas, Amyn A. Malik, Tianyang Mao, Anjelica Martin, Irene Matos, David McDonald, Maksym Minasyan, Adam J. Moore, M. Catherine Muenker, Maura Nakahata, Nida Naushad, Allison Nelson, Jessica Nouws, Angela Nunez, Marcella Nunez-Smith, Abeer Obaid, Camila Odio, Ji Eun Oh, Saad Omer, Isabel M. Ott, Annsea Park, Hong-Jai Park, Xiaohua Peng, Sarah Prophet, Harold Rahming, Tyler Rice, Aaron Ring, Kadi-Ann Rose, Lorenzo Sewanan, Lokesh Sharma, Albert Shaw, Denise Shepard, Erin Silva, Julio Silva, Mikhail Smolgovsky, Eric Song, Nicole Sonnert, Yvette Strong, Codruta Todeasa, Maria Tokuyama, Jordan Valdez, Sofia Velazquez, Arvind Venkataraman, Pavithra Vijayakumar, Eric Y. Wang, Anne E. Watkins, Elizabeth B. White, Patrick Wong, and Yexin Yang.
