# Supplementary figures and images for: Sequencing SARS-CoV-2 genomes from saliva
Source: Virus Evol. 2022 Jan 3;8(1):veab098. doi: 10.1093/ve/veab098 (PMC9074962; doi:10.1093/ve/veab098)

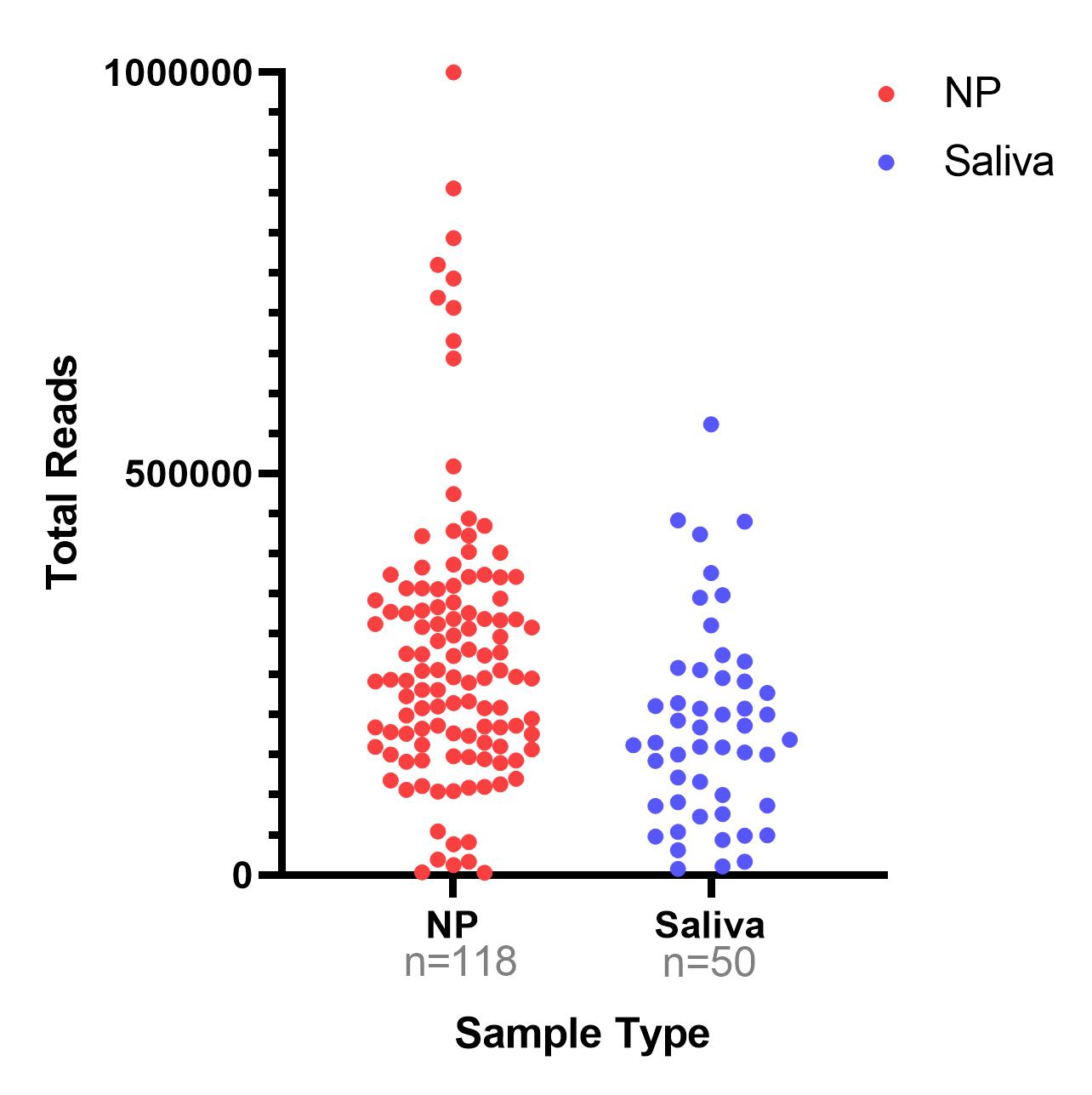

Supplement: veab098_Supp [file veab098_supp.zip › Supplemental Figure 1.jpg]

Pair01

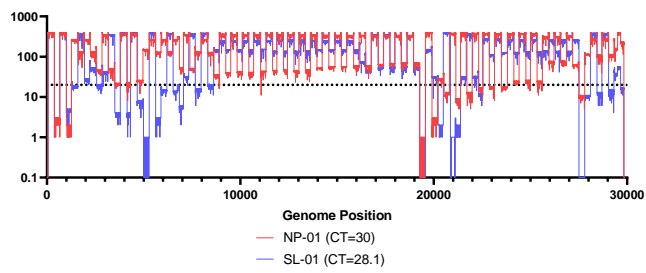

Pair06

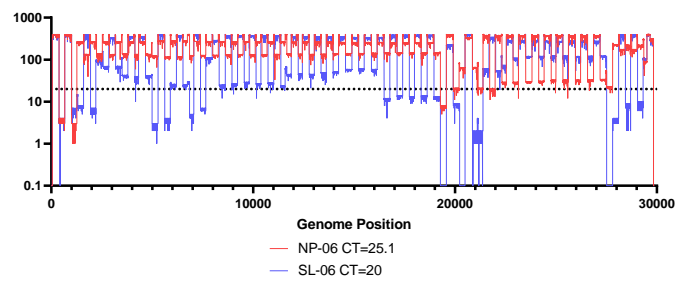

Pair02

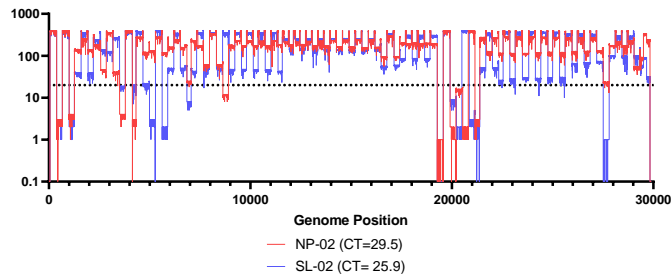

Pair07

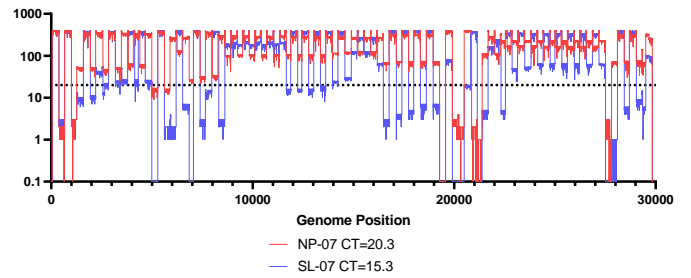

Pair03

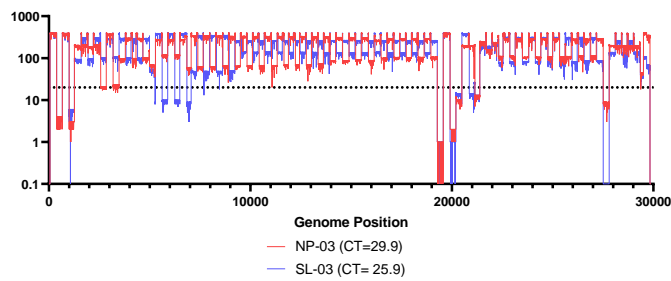

Pair08

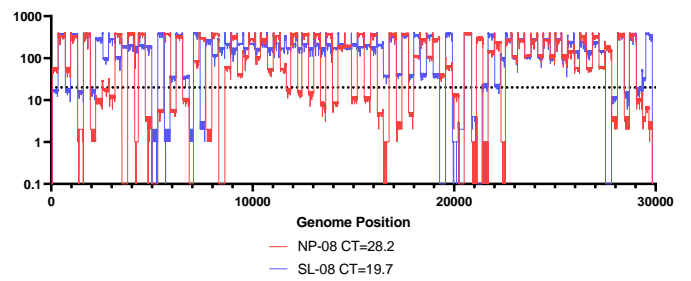

Pair04

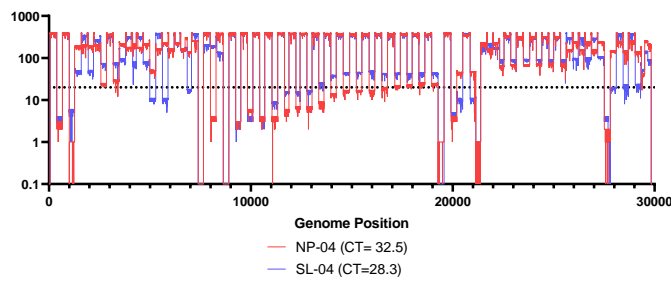

Pair09

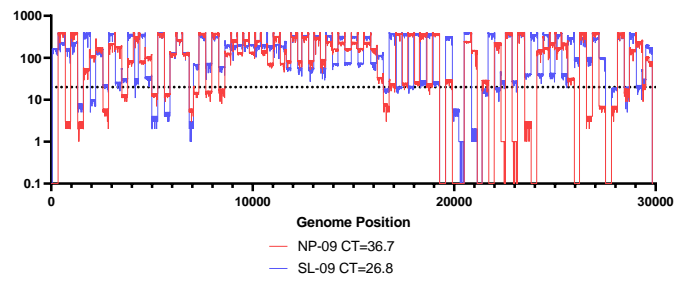

Pair05

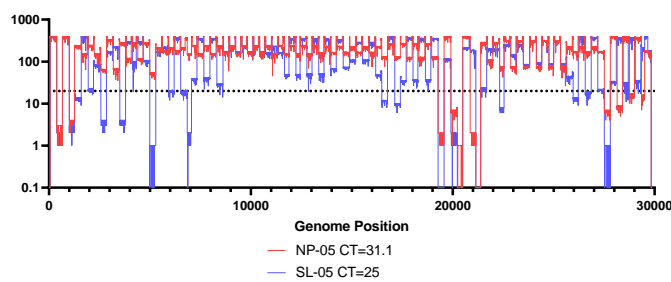

Pair12

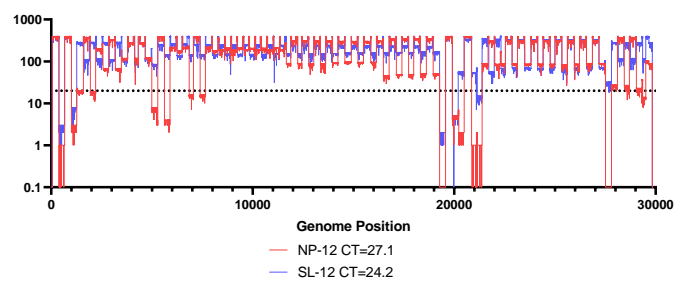

Supplement: veab098_Supp [file veab098_supp.zip › Supplemental Figure 2.pdf]
